# Healthcare disparities among orthopedic trauma patients in the USA: socio-demographic factors influence the management of calcaneus fractures

**DOI:** 10.1186/s13018-019-1402-8

**Published:** 2019-11-12

**Authors:** Boris A. Zelle, Nicolas A. Morton-Gonzaba, Christopher F. Adcock, John V. Lacci, Khang H. Dang, Ali Seifi

**Affiliations:** 1 Department of Orthopaedic Surgery, UT Health San Antonio, 7703 Floyd Curl Dr, MC-7774, San Antonio, TX 78229 USA; 2 Department of Neurosurgery-Neuro Critical Care, UT Health San Antonio, San Antonio, TX USA

**Keywords:** Calcaneus, Fracture, Open reduction internal fixation, Trends, Socioeconomic

## Abstract

**Background:**

Socio-demographic factors have been suggested to contribute to differences in healthcare utilization for several elective orthopedic procedures. Reports on disparities in utilization of orthopedic trauma procedures remain limited. The purpose of our study is to assess the roles of clinical and socio-demographic variables in utilization of operative fixation of calcaneus fractures in the USA.

**Methods:**

The National Inpatient Sample (NIS) dataset was used to analyze all patients from 2005 to 2014 with closed calcaneal fractures. Multivariate logistic regression analyses were performed to evaluate the impact of clinical and socio-demographic variables on the utilization of surgical versus non-surgical treatment.

**Results:**

A total of 17,156 patients with closed calcaneus fractures were identified. Operative treatment was rendered in 7039 patients (41.03%). A multivariate logistic regression demonstrated multiple clinical and socio-demographic factors to significantly influence the utilization of surgical treatment including age, gender, insurance status, race/ethnicity, income, diabetes, peripheral vascular disease, psychosis, drug abuse, and alcohol abuse (*p* <  0.05). In addition, hospital size and hospital type (teaching versus non-teaching) showed a statistically significant difference (*p* <  0.05).

**Conclusions:**

Besides different clinical variables, we identified several socio-demographic factors influencing the utilization of surgical treatment of calcaneus fractures in the US patient population. Further studies need to identify the specific patient-related, provider-related, and system-related factors leading to these disparities.

## Background

The treatment of calcaneus fractures remains challenging and controversial. Historically, these injuries were managed non-operatively given the high concern for postoperative morbidity [1]. Over the past decades, improvements in diagnostic imaging, surgical techniques, understanding of the soft tissue injury, and advances in surgical implants have improved clinical outcomes [2–4]. Open reduction and internal fixation remains a standard treatment option for displaced intraarticular calcaneus fractures [3, 4]. Despite these improvements, the risk of soft tissue complications remains relatively high and patients with certain risk factors, such as smoking and diabetes, have been suggested to be unfavorable candidates for this procedure [5]. In addition, the question remains to what extent different socio-demographic factors, such as race, insurance status, and income, may potentially contribute to differences in healthcare utilization of surgical fixation of calcaneus fractures.

Recent reports have emphasized significant ethnic and racial disparities in utilization of elective orthopedic procedures, such as arthroplasty, carpal tunnel release, and lumbar spinal fusions [6–9]. In particular, ethnic and racial minorities seem to be less likely to utilize elective orthopedic procedures. The exact etiology for these healthcare disparities is not fully known. Most likely, the underlying reasons for these findings are multifactorial and may include patient-related factors as well as provider-related factors. In addition, system-related factors, such as access to healthcare, may further contribute to this phenomenon [7, 10–13]. Most of the data on ethnic and racial disparities regarding utilization of orthopedic procedures refers to elective orthopedic procedures. Regarding orthopedic trauma procedures, a recent report suggested increased rates of fixation of clavicle fractures in Caucasians and patients of higher socioeconomic status [14]. However, studies from the orthopedic trauma literature depicting healthcare utilization disparities remain limited.

The goal of our study is to further assess the roles of socio-demographic, specifically race, and other clinical variables in operative fixation of calcaneus fractures in the USA. We hypothesize that several socio-demographic variables are associated with decreased rates of fixation of these fractures.

## Methods

### Database

The Agency for Healthcare Research and Quality developed the National Inpatient Sample (NIS), which is a part of the Healthcare Cost and Utilization Project (HCUP). The NIS database is publicly available and is comprised of an approximately 20% stratified sample of all discharges at US hospitals, excluding rehabilitation and long-term acute care facilities. Information includes diagnosis/procedure codes (International Classification of Disease version 9 Clinical Modification, ICD-9 CM; Current Procedure Terminology, CPT), demographic data, and insurance. This database was chosen because it allowed regression analyses of treatment trends and demographic data. The entry input of patient’s comorbidities has shown to be accurate through previous studies and has been tested through the Elixhauser Comorbidity Software [15]. The study was determined to be non-regulated research and did not qualify for review by the Institutional Review Board (IRB) of our institution.

### Patients

From the 2005–2014 NIS database sets, all discharges with a closed calcaneus fracture (code 825.0) were included in this study. Observations with the ICD-9 CM codes 825.1; 825.20–825.29; 825.30–825.39 were excluded from this study as they code for other foot fractures. Since other foot fractures were excluded, surgical fixation of calcaneus fracture was identified through the ICD-9 CM code 79.37. Patients with missing data were excluded. Several potentially confounding clinical and socio-demographic variables were obtained from the database and tested for any differences between the operative and non-operative group. The socio-demographic variables included age, sex, race/ethnicity, estimated median household income quartile based on zip code, and insurance status. Potentially confounding comorbidities included diabetes mellitus, acquired immune deficiency syndrome (AIDS), illicit drug abuse, peripheral vascular diseases, smoking status, alcohol abuse, obesity, psychosis, and depression. Additional potentially confounding variables included hospital type (teaching versus non-teaching) and hospital size, as divided by number of beds into small, medium, and large.

### Statistical analysis

The statistical analyses were performed on a total of 17,156 discharged patients who were identified by the inclusion and exclusion criteria as above. All statistical analyses were performed using Stata 15.1 (StataCorp, College Station, TX, USA). All continuous variables were tested for normal distribution. Normally distributed data was reported as means with standard deviation (SD). Not normally distributed data was reported as median with range. The univariate analysis used *T* tests for continuous variables and chi-square testing for non-continuous variables. All clinical and socio-demographic variables were tested for statistical significance in a univariate analysis. All clinical and socio-demographic variables that demonstrated statistical significance at the level of *p* <  0.05 in the univariate analysis were enrolled into the multivariate regression analysis. A multivariate regression model was created by including all statistically significant clinical and socio-demographic variables found in the univariate analysis. The independent variables included in the multivariate regression analysis were age, sex, race/ethnicity, estimated median household income, insurance status, diabetes mellitus (complicated and uncomplicated), AIDS, illicit drug abuse, peripheral vascular diseases, alcohol abuse, obesity, psychosis, and depression. In addition, hospital size (by number of beds) and type of hospital (teaching versus non-teaching) were included in the multivariate regression. Utilization of open reduction and internal fixation of calcaneus fracture was the dependent variable.

## Results

Between 2005 and 2014, a total of 17,156 patients were included for participation in this study. The socio-demographic data of our patient sample is shown in Table 1. 10,117 patients underwent non-operative management of their calcaneus fracture while 7039 patients underwent open reduction and internal fixation. The univariate analysis suggested that as compared to the operative group, patients who were treated non-operatively were more likely to be older and female (*p* <  0.001). Race and ethnicity were significantly different between the non-operative and the operative group (*p* <  0.001), whereby the rates of surgical fixation were lower among African-American and Hispanic patients. In addition, the insurance status, estimated median household income based on zip code, hospital type (teaching versus non-teaching), and hospital size showed statistically significant differences in the univariate analysis (*p* <  0.001). Moreover, we identified several clinical variables that were associated with a significantly lower rate of surgical fixation as per the univariate analysis (Table 2). These clinical variables included diabetes mellitus (complicated and uncomplicated), AIDS, illicit drug abuse, peripheral vascular diseases, alcohol abuse, psychosis, depression, and obesity (*p* <  0.05).

**Table 1:** Socio-demographic variables in patients with calcaneus fractures, univariate comparison between non-operative and operative treatment

	Total	Non-operative	Operative	*p*
Sample size	17,156	10,117 (58.97%)	7039 (41.03%)	
Age (mean ± SD), years	47.79 ± 17.13	49.20 ± 18.48	45.78 ± 14.75	< 0.001
Female, *n* (%)	5649	3756 (37.13%)	1893 (26.89%)	< 0.001
Payer, *n* (%)				< 0.001
Medicare	3043	2230 (73.28%)	813 (26.72%)	
Medicaid	2027	1284 (63.34%)	743 (36.66%)	
Private payer	6860	3656 (53.29%)	3204 (46.71%)	
Self-payer	2225	1391 (62.51%)	834 (37.49%)	
No charge	288	158 (54.86%)	130 (45.14%)	
Other	2713	1398 (51.52%)	1315 (48.48%)	
Race/ethnicity, *n* (%)				< 0.001
White	12,693	7419 (58.44%)	5274 (41.46%)	
African-American	1465	971 (66.27%)	494 (33.73%)	
Hispanic	1927	1152 (59.78%)	775 (40.22%)	
Asian	360	186 (51.66%)	174 (48.34%)	
Native American	112	73 (65.17%)	39 (34.83%)	
Other	599	316 (52.75%)	283 (47.25%)	
Income*, *n* (%)				< 0.001
0–24th percentile	4889	3099 (63.38%)	1790 (36.62%)	
25–49th percentile	4363	2578 (59.08%)	1785 (40.92%)	
50–74th percentile	4221	2418 (57.28%)	1803 (42.72%)	
75–100th percentile	3683	2022 (54.90%)	1661 (45.10%)	
Teaching hospital/location, *n* (%)				< 0.001
Rural	1241	850 (68.49%)	391 (31.51%)	
Urban non-teaching	5430	3149 (57.99%)	2281 (42.01%)	
Urban teaching	10,299	6007 (58.32%)	4292 (41.68%)	
Hospital size (number of beds)				0.036
Small	1833	1046 (57.06%)	787 (42.93%)	
Medium	4065	2457 (60.44%)	1608 (39.56%)	
Large	11,072	6503 (58.73%)	4569 (41.27%)

**Table 2:** Clinical variables in patients with calcaneus fractures, univariate comparison between non-operative and operative treatment

	Total	Non-operative	Operative	*p*
Sample size	17,156	10,117 (58.97%)	7039 (41.03%)	
Diabetes, uncomplicated	1386	936 (67.53%)	450 (32.47%)	< 0.001
Diabetes, with chronic complications	495	392 (79.19%)	103 (20.81%)	< 0.001
AIDS	32	25 (78.13%)	7 (21.88%)	0.026
Drug abuse	1076	798 (74.16%)	278 (25.83%)	< 0.001
Peripheral vascular disorders	656	483 (73.63%)	173 (26.37%)	< 0.001
Alcohol abuse	1333	1024 (76.82%)	309 (23.18%)	< 0.001
Psychosis	151	114 (75.50%)	37 (24.50%)	< 0.001
Depression	1271	824 (64.83%)	447 (35.17%)	< 0.001
Obesity	789	514 (65.15%)	275 (34.85%)	< 0.001
Smoking	4213	2490 (59.10%)	1723 (40.90%)	0.85

The multivariate logistic regression demonstrated that several clinical and socio-demographic variables significantly influenced the type of treatment (Table 3). The socio-demographic variables associated with a significantly lower utilization of open reduction and internal fixation included older age, female gender, Medicare, minority status (African Americans and Hispanics), and lower estimated income by zip code (*p* <  0.05). Clinical comorbidities associated with a significantly lower utilization of surgical treatment included diabetes mellitus (complicated and uncomplicated), illicit drug abuse, peripheral vascular diseases, alcohol abuse, and psychosis (*p* <  0.05). In addition, it was recorded that the rates of surgical fixation were lower at rural hospitals as well as hospitals with a smaller number of beds (*p* <  0.05).

**Table 3:** Multivariate logistic regression

	Odds ratio	95% confidence interval		*p*
Age	0.994	0.992	0.997	< 0.001
Female	0.692	0.643	0.745	< 0.001
Payer, (*base—Medicare*)				
Medicaid	1.352	1.172	1.559	< 0.001
Private payer	1.746	1.565	1.949	< 0.001
Self-payer	1.224	1.064	1.409	0.005
No charge	1.688	1.296	2.199	< 0.001
Other	1.717	1.509	1.955	< 0.001
Race, (*base—White*)				
African-American	0.807	0.714	0.911	0.001
Hispanic	0.895	0.806	0.993	0.038
Asian	1.296	1.043	1.611	0.019
Native American	0.867	0.566	1.329	0.514
Other	1.160	0.977	1.376	0.089
Income (base—0–24th percentile)				
25–49th percentile	1.106	1.013	1.208	0.025
50–74th percentile	1.137	1.039	1.245	0.005
75–100th percentile	1.233	1.121	1.356	< 0.001
Teaching hospital/location (base—urban non-teaching)				
Rural	0.657	0.572	0.754	< 0.001
Urban teaching	0.979	0.913	1.050	0.565
Hospital size (base—small)				
Medium	0.874	0.778	0.982	0.024
Large	0.919	0.828	1.020	0.114
Comorbidities				
Diabetes, uncomplicated	0.802	0.708	0.909	0.001
Diabetes, with chronic complications	0.536	0.426	0.676	< 0.001
AIDS	0.585	0.243	1.408	0.232
Drug abuse	0.567	0.488	0.660	< 0.001
Peripheral vascular	0.401	0.297	0.540	< 0.001
Alcohol abuse	0.414	0.361	0.476	< 0.001
Psychosis	0.728	0.612	0.866	< 0.001
Depression	0.961	0.847	1.091	0.547
Obesity	0.976	0.832	1.146	0.775

## Discussion

While healthcare utilization disparities have been shown in several areas of orthopedics, such as arthroplasty, upper extremity, and spinal surgeries, the literature has been limited within the field of orthopedic trauma [6, 8, 16]. Our study showed that several factors influence the treatment of closed calcaneus fractures in the US patient population. Expectedly, utilization of open reduction and internal fixation was influenced by medical comorbidities including diabetes, peripheral vascular disease, history of alcohol abuse, history of drug abuse, and psychoses. In addition, it is an expected finding that the rates of surgical treatment were lower at small hospitals and non-teaching facilities. Interestingly, we were able to identify specific socio-demographic factors with a significant influence on utilization of open reduction and internal fixation of calcaneus fractures. Thus, African-American patients and Hispanic patients with calcaneus fractures were less likely to undergo open reduction and internal fixation as compared to Caucasian patients, whereas Asian patients with calcaneus fractures seemed more likely to undergo surgery. In addition, insurance status and median zip code income showed a significant influence on the type of treatment.

Our study has both strengths and limitations. The data presented in this study demonstrates socio-demographic disparities regarding utilization of surgical fixation of calcaneus fractures. However, we do not have any research data to explain the exact reasons for these findings. Thus, future investigations may address specific patient-related, provider-related, and system-related factors contributing to this phenomenon. In addition, our data was retrieved from a multicenter database. This is inherently associated with certain limitations, such as variable data entry. In addition, the database used in this study is limited to inpatient admissions and does not account for those patients with calcaneus fractures treated on an outpatient basis. This certainly introduces a potential selection bias due to an over-representation of patients with potentially more comorbidities and social issues. Moreover, we were unable to collect fracture-specific information, such as fracture classification and the associated soft tissue injury. We identified patients with the diagnosis of a closed calcaneus fracture as per ICD-9 code, and we do not have any radiographic data to record fracture displacement, comminution, or intraarticular involvement. However, with regard to our main study question of racial and social disparities, we assume that the severity of fractures was equally distributed among patients of different race and socioeconomic status. We, therefore, feel that the lack of radiologic data did not introduce any significant bias into the study with regard to our main results.

Racial and social healthcare disparities have been identified within different elective areas of orthopedic surgery, such as hip and knee arthroplasty, shoulder arthroplasty, ankle arthroplasty, spinal fusion, cubital and carpal tunnel surgery, elective hardware removal following pediatric femoral shaft fractures, and treatment of meniscal tears [6–9, 13, 16–22]. In particular, minorities were significantly less likely to undergo these elective orthopedic procedures. Regarding healthcare disparities in orthopedic trauma patients, a recent report by Schairer et al. [14] suggested increased rates of fixation of clavicle fractures in Caucasians and patients of higher socioeconomic status. However, to our best knowledge, reports on racial and social disparities regarding utilization of orthopedic trauma procedures remain limited. We can only speculate on why these healthcare disparities have gained little attention in the orthopedic trauma literature. As a possible explanation, we suggest that there may be the common misconception that in orthopedic trauma, the decision of operative versus non-operative treatment may be solely based on the injury pattern and the actual fracture pathology. However, the role of open reduction and internal fixation in the treatment of calcaneus fractures is not yet fully defined, and non-operative treatment frequently remains a feasible treatment option [2]. Thus, the indication for open reduction and internal fixation in calcaneus fractures depends on various factors. Besides the fracture pattern, the patient’s surgical risk factors, such as smoking and diabetes, play a significant role in the decision making [5]. Given the relatively high risk of soft tissue complications associated with this surgical procedure, careful risk/benefit discussions frequently play a predominant role in the shared decision-making process. This complex decision-making process is certainly influenced by patients’ preferences as well as surgeons’ preferences, which potentially may be associated with the risk of introducing bias based on the patient’s socio-demographics.

While our study demonstrated socio-demographic disparities regarding utilization of open reduction and internal fixation of calcaneus fractures, the exact reasons for these disparities remain unclear. Examining the exact causation for these findings observed in our study is beyond the scope of the current analysis. Data from elective orthopedic specialties has suggested various patient-related, provider-related, and system-related factors which may play a role for racial and social disparities regarding utilization of orthopedic procedures. With regard to patient-related factors, different studies have shown that African-American patients are less willing to consider surgical treatment options for their hip and knee osteoarthritis [11, 23–25]. In addition, provider-related factors have been suggested to contribute to racial and social disparities in orthopedic surgery. Thus, it has been reported that orthopedic providers have less relationship building communications with African-American patients as compared to white patients [26]. Moreover, system-related factors, such as access to healthcare, may contribute to these disparities. Jancuska et al. [8] reported that Medicaid patients and minorities were less likely to undergo spinal fusion at high-volume centers suggesting access issues for these vulnerable populations. Also, Bach et al. [27] reported significant difficulty for providers to access resources for African Americans, including subspecialty referrals, diagnostic imaging, hospital admission, and high-quality ancillary services. Although this data from elective orthopedic procedures cannot be extrapolated to orthopedic trauma patients, we suggest that the racial and social disparities observed in our study are multifactorial and may include various patient-related, provider-related, and system-related factors.

## Conclusions

In conclusion, our study demonstrated the significant influence of clinical and socio-demographic variables on utilization of open reduction and internal fixation of calcaneus fractures in the US patient population. Besides the influence of several clinical comorbidities, our study showed decreased utilization for minorities, patients from lower income zip codes, and patients without private insurance. Future studies may investigate the specific patient-related, provider-related, and system-related factors that are contributing to healthcare disparities within orthopedic trauma surgery.

## Data Availability

The datasets generated and/or analyzed during the current study are publicly available.

## Abbreviations

AIDS: Acquired immune deficiency syndrome
CPT: Current Procedure Terminology
HCUP: Healthcare Cost and Utilization Project
ICD-9: International Classification of Disease version 9
IRB: Institutional Review Board
NIS: National Inpatient Sample
SD: Standard deviation
